# Hippo signaling regulates spontaneous human trophoblast syncytialization in three-dimensional cultures

**DOI:** 10.1093/molehr/gaag054

**Published:** 2026-09-08

**Authors:** Michael Zheng, Lin Zhao, Richard Xi, Jahdiel DeNobrega, Mary Tammo, Stephen J Renaud, Peeyush K Lala

**Affiliations:** Department of Anatomy and Cell Biology, Schulich School of Medicine and Dentistry, University of Western Ontario, London, ON, Canada; Department of Anatomy and Cell Biology, Schulich School of Medicine and Dentistry, University of Western Ontario, London, ON, Canada; Department of Anatomy and Cell Biology, Schulich School of Medicine and Dentistry, University of Western Ontario, London, ON, Canada; Department of Anatomy and Cell Biology, Schulich School of Medicine and Dentistry, University of Western Ontario, London, ON, Canada; Department of Anatomy and Cell Biology, Schulich School of Medicine and Dentistry, University of Western Ontario, London, ON, Canada; Department of Anatomy and Cell Biology, Schulich School of Medicine and Dentistry, University of Western Ontario, London, ON, Canada; Children’s Health Research Institute, London Health Sciences Centre Research Institute, London, ON, Canada; Department of Anatomy and Cell Biology, Schulich School of Medicine and Dentistry, University of Western Ontario, London, ON, Canada; Children’s Health Research Institute, London Health Sciences Centre Research Institute, London, ON, Canada

**Keywords:** human placenta, trophoblast stem cells, 3D culture, syncytiotrophoblast, trophoblast differentiation, Hippo signaling, YAP1/TAZ, organoid

## Abstract

Proper trophoblast development is essential for placental function and is a key determinant of pregnancy outcomes. Three-dimensional spheroid and organoid cultures using cells from placental tissues or trophoblast stem cells have emerged as models that recapitulate cell–cell interactions and aspects of the physiological environment. Trophoblast organoids typically contain self-renewing cytotrophoblasts located toward the periphery in contact with basement membrane, whereas cells within the core fuse into hormone-producing syncytiotrophoblasts. Removing cells from matrix and/or culturing them in suspension can reverse this organization. In both cases, spontaneous syncytialization occurs, but the underlying mechanisms remain poorly understood. To identify signaling pathways associated with spontaneous trophoblast differentiation in three-dimensional conditions, human trophoblast stem cells were either maintained in the stem state as two-dimensional (2D) monolayers or aggregated in suspension without an exogenous matrix to form three-dimensional cultures. Expression and localization of trophoblast subpopulation markers were compared between two- and three-dimensional cultures. Three-dimensional cultures displayed increased spontaneous differentiation into the syncytiotrophoblast lineage along the periphery, accompanied by reduced proliferation of cytotrophoblasts. Consistent with this observation, BeWo choriocarcinoma cells exhibited increased chorionic gonadotropin levels in three-dimensional culture, whereas this response was not observed in HTR8/SVneo immortalized trophoblasts. Gene expression and pathway enrichment analyses identified Hippo signaling as altered in three-dimensional trophoblast stem cell cultures, with reduced expression of several genes associated with YAP1/TAZ-TEAD transcriptional activity. Inhibition of LATS1/2, which activates YAP1/TAZ-TEAD signaling, reduced spontaneous syncytiotrophoblast formation. These findings suggest that reduced YAP1/TAZ-TEAD activity may facilitate self-organized syncytialization in three-dimensional trophoblast cultures.

## Graphical abstract

**Graphical Abstract:**
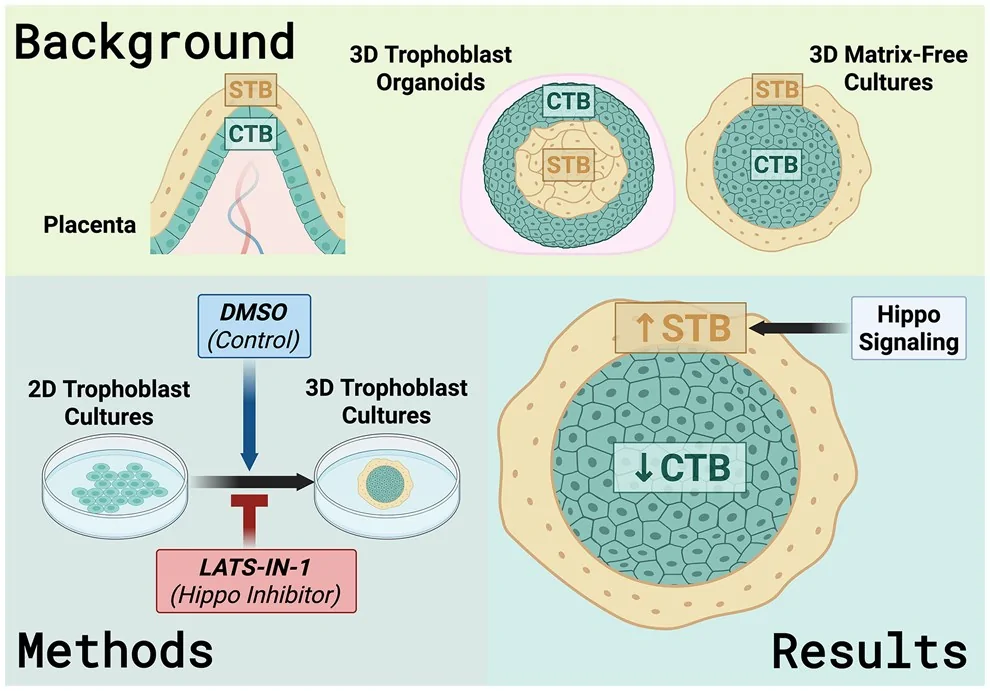
**Trophoblast subpopulations and Hippo signalling in three-dimensional matrix-free cultures.** This study compares cytotrophoblast (CTB) and syncytiotrophoblast (STB) subpopulations between two-dimensional (2D) and three-dimensional (3D) cultures. Created in BioRender. Zheng, M. (2025) https://BioRender.com/8w76tb1.

## Introduction

The placenta is a temporary yet essential organ that forms during pregnancy to support fetal growth and development. It performs multiple functions required for pregnancy maintenance and fetal growth, such as promoting nutrient, gas, and waste transfer between maternal and fetal blood, as well as hormone production and metabolism (Turco and Moffett, 2019). These functions are primarily performed by trophoblasts, which are placental epithelial cells derived initially from the trophectoderm of preimplantation embryos. Trophoblast lineages originate from progenitor cytotrophoblasts (CTBs) that differentiate bidirectionally towards syncytiotrophoblast (STB) and extravillous trophoblast (EVT) pathways. The STB is a multinucleated entity formed and replenished by the fusion of CTBs, which erodes uterine tissue after implantation and ultimately lines the outer surfaces of placental villi in direct contact with maternal blood. Key functions of the STB include the exchange of nutrients, gases, wastes, and other molecules between maternal and fetal circulations, the production of hormones and other substances needed for maternal-fetal communication, and the formation of a barrier to prevent potentially harmful substances from accessing fetal blood (Napso *et al.*, 2018; Schust *et al.*, 2021; Renaud and Jeyarajah, 2022). At the distal tips of the large anchoring villi in proximity to the decidua (modified uterine endometrium), CTBs preferentially differentiate into EVTs. This transformation into EVTs involves proliferation into stratified cell columns, followed by a gradual epithelial-to-mesenchymal transition, allowing them to invade the decidua up to the inner third of the myometrium. A subset of EVTs displaces the endothelium and smooth muscle cells surrounding uterine blood vessels, resulting in a steady maternal blood flow towards the placenta and ensuring fetal sustenance (Lala and Nandi, 2016). Dysregulated trophoblast differentiation is implicated in pregnancy complications such as preeclampsia and fetal growth restriction, which can have lasting consequences for both pregnant individuals and their children (Redman, 1991).

To advance the study of human trophoblast biology, many *in vitro* models have emerged to meet this pressing demand. These include widely-used trophoblast cell lines such as BeWo choriocarcinoma cells (Pattillo and Gey, 1968) and immortalized first-trimester HTR-8/SVneo (HTR) trophoblasts (Graham *et al.*, 1993). While these cell lines do not fully recapitulate the phenotype of primary trophoblasts, BeWo cells are adept at modelling STB formation through activation of the cAMP pathway (Friedman and Skehan, 1979), and HTRs exhibit many EVT-associated activities (Iacob *et al.*, 2008; Lala *et al.*, 2012; Halari *et al.*, 2020). A recent breakthrough in this field was the derivation and culture of human trophoblast stem (TS) cells from first-trimester placentas and blastocysts (Okae *et al.*, 2018). Human TS cells are capable of long-term self-renewal as CTBs through the activation of Wnt and epidermal growth factor (EGF) signaling, along with inhibition of histone deacetylase activity and transforming growth factor β (TGFβ) signaling. Human TS cells can be differentiated into STB or EVTs by removing EGF, Wnt agonists, and histone deacetylase inhibitors (among other factors) along with adding the cAMP agonist, forskolin (for STB differentiation); or by further inhibiting the TGFβ pathway and adding neuregulin and basement membrane extract (for EVT differentiation).

Another approach that has recently gained traction is the use of three-dimensional (3D) cultures such as organoids, which recapitulate key aspects of tissue architecture and cellular organization found in native tissues (Kretzschmar and Clevers, 2016). The pioneering trophoblast organoids were developed by embedding CTBs isolated from first-trimester placentas in Matrigel domes (Haider *et al.*, 2018; Turco *et al.*, 2018). Trophoblast organoids generated in this way maintain layers of CTB near their periphery (adjacent to the Matrigel) and spontaneously form STB within their core. Trophoblast organoids have also been derived from human TS cells (Saha *et al.*, 2020; Sheridan *et al.*, 2021; Karvas *et al.*, 2022; Shannon *et al.*, 2022, 2024; Wu *et al.*, 2023), pluripotent stem cells (Castel *et al.*, 2020; Io *et al.*, 2021; Karvas *et al.*, 2022), and late-gestation placental tissues (Yang *et al.*, 2022), and typically display this localization of CTBs at the periphery adjacent to the Matrigel and STB toward the lumen.

Recently, it was shown that removing trophoblast organoids from Matrigel promotes a more physiologically relevant organization, with CTBs positioned internally and STB forming along the outer surface (Yang *et al.*, 2024). A similar organization of CTB and STB subpopulations develops when human TS cells are grown in microwells without Matrigel, suggesting that extracellular matrix components may provide cues that direct STB differentiation (Hori *et al.*, 2024). Furthermore, culturing human TS cells on collagen-coated beads that simulate the basement membrane promotes a similar phenotype of CTBs and STBs in the resulting 3D cultures (Zhou *et al.*, 2025). While employing different techniques, this collective evidence complements those of our earlier findings, which involves the aggregation of human TS cells into 3D matrix-free cultures followed by suspension culturing (Jeyarajah *et al.*, 2022). These 3D cultures spontaneously form STB at their periphery akin to the proper anatomical configuration of a placental villus; however, the mechanisms driving this transformation remain unknown.

The spatial context of 3D cultures may be akin to that of *in situ* trophoblast development, where multiple signaling pathways converge to establish cell specification and polarity. One such example is the Hippo pathway, which regulates organ size (Camargo *et al.*, 2007; Dong *et al.*, 2007), embryonic development (Saha *et al.*, 2020), and cancer growth (Lv and Zhou, 2023). The Hippo pathway regulates the activation of transcriptionally enhanced associated domain (TEAD) transcription factors (TEAD1-4), which requires binding with co-activators such as the yes-associated protein 1 (YAP1) or transcriptional co-activator with PDZ binding motif (TAZ). Activating the Hippo pathway triggers a kinase cascade involving large tumour suppressor kinase 1/2 (LATS1/2), which prevents the nuclear translocation of YAP1 and TAZ. Within trophoblasts, the Hippo pathway plays a multifaceted role (Soncin and Parast, 2020). In preimplantation embryos, activation of TEAD transcription factor 4 (TEAD4) directs cells towards trophectoderm formation along the periphery (Nishioka *et al.*, 2008). In first-trimester placentas and human TS cells, TEAD4 (Saha *et al.*, 2020; Mizutani *et al.*, 2022) and YAP1 (Meinhardt *et al.*, 2020) are essential for promoting CTB self-renewal and inhibiting STB formation. Conversely, expression of the gene encoding TAZ, *WWTR1*, has been linked to cell proliferation and EVT differentiation (Ray *et al.*, 2022). Ultimately, the role of Hippo pathway signaling in 3D trophoblast cultures remains largely unexplored. In the current study, we investigated the potential of 3D matrix-free human TS cultures to generate trophoblast subpopulations and examined the potential role of Hippo pathway-associated signaling in this process. We show that 3D matrix-free culture promotes spontaneous outward differentiation of human TS cells primarily toward the STB lineage, while activation of YAP1/TAZ-TEAD signaling through pharmacological LATS1/2 inhibition reduces spontaneous STB formation.

## Materials and methods

### 2D cell culture

All cells were cultured in a humidified incubator at 37°C with 5% CO_2_ and routinely assessed for mycoplasma contamination.

Human TS cells were initially isolated from first-trimester human placentas. Experiments were performed with the CT27 (female, 7-week) line generously gifted by Hiroaki Okae and Takahiro Arima (Tohoku University Graduate School of Medicine, Sendai, Japan) and cultured under previously reported conditions (Okae *et al.*, 2018). Basal TS media contained DMEM/F12 media (10-090-CV, Corning Inc., Corning, NY, USA) supplemented with 0.2% (v/v) embryonic stem cell fetal bovine serum (FBS, 16141061, Thermo Fisher Scientific, Mississauga, ON, Canada), 50 U/ml penicillin, 50 µg/ml streptomycin, 0.1 mM 2-mercaptoethanol (21985-023, Thermo Fisher Scientific), 0.3% bovine serum albumin (A1595/A9205, Sigma-Aldrich, Oakville, ON, Canada), 1% insulin-transferrin-selenium-ethanolamine (51500-056, Thermo Fisher Scientific), 50 ng/ml EGF (E9644, Sigma-Aldrich), and 1.5 µg/ml L-ascorbic acid (A8960, Sigma-Aldrich). Human TS cells were cultured in complete TS media, which is basal TS media further supplemented with 5 µM Y27632 (1293823, Biogems, Westlake Village, CA, USA), 0.5 µM A83-01 (9094360, Biogems), 2 µM CHIR99021 (2520691, Biogems), 1 µM SB431542 (3014193, Biogems), and 0.8 mM valproic acid (P4543, Sigma-Aldrich). Before reaching 90% confluency, human TS cells were passaged using TrypLE (12604013, Thermo Fisher Scientific) or Accutase (07920, STEMCELL Technologies, Vancouver, BC, Canada; A1110501, Thermo Fisher Scientific) and were not maintained beyond 20 consecutive passages.

Our laboratory previously immortalized a first-trimester primary human trophoblast cell line, HTR-8, through transfection with a plasmid encoding the simian virus 40 large T antigen as the HTR-8/SVneo cell line (herein referred to as HTR cells) (Graham *et al.*, 1993). HTR cells have been distributed internationally and are used frequently as a model of first-trimester EVTs (available through American Type Culture Collection, CRL-3271). Karyotyping and RNA-sequencing of HTRs reveal that this is a female cell line (Drennan *et al.*, 2010; Lapehn *et al.*, 2025). HTR cells were cultured in RPMI-1640 medium (R8758, Sigma-Aldrich) supplemented with 10% FBS (12483020, Thermo Fisher Scientific), 100 U/ml penicillin, and 100 µg/ml streptomycin. Before reaching 90% confluency, HTR cells were passaged through trypsinization and were not maintained beyond 20 consecutive passages.

BeWo cells were originally derived from a human metastatic choriocarcinoma (available through American Type Culture Collection, CCL-98). They were cultured in DMEM/F12 media (10-090-CV, Corning Inc.) supplemented with 10% FBS (12483020, Thermo Fisher Scientific), 100 U/ml penicillin, and 100 µg/ml streptomycin. Before reaching 90% confluency, BeWo cells were passaged through trypsinization and were not maintained beyond 20 consecutive passages.

For two-dimensional (2D) cultures, human TS and HTR cells were plated onto cell culture dishes coated with 5 µg/ml collagen IV (3410-010-02, Bio-Techne R&D Systems, Minneapolis, MN, USA) under complete TS media conditions and collected after 2 days. BeWo cells were plated in cell culture dishes not precoated with collagen and grown in BeWo media.

### Differentiation in 2D cultures

Human TS cells were differentiated into STB or EVTs on cell culture dishes coated with 2.5 µg/ml or 1 µg/ml collagen IV, respectively, for up to 5 days under previously reported conditions (Okae *et al.*, 2018).

For STB differentiation, human TS cells were cultured in DMEM/F12 media supplemented with 4% (v/v) knockout serum replacement (10828-010, Thermo Fisher Scientific), 50 U/ml penicillin, 50 µg/ml streptomycin, 0.1 mM 2-mercaptoethanol, 0.3% bovine serum albumin, 1% insulin-transferrin-selenium-ethanolamine, 2.5 µM Y27632, and 2 µM forskolin (F6886, Sigma-Aldrich).

For EVT differentiation, human TS cells were cultured in DMEM/F12 media supplemented with 4% (v/v) knockout serum replacement, 50 U/ml penicillin, 50 µg/ml streptomycin, 0.1 mM 2-mercaptoethanol, 0.3% bovine serum albumin, 1% insulin-transferrin-selenium-ethanolamine, 2.5 µM Y27632, and 7.5 µM A83-01. Cultrex basement membrane extract (3432-010-01, Bio-Techne R&D Systems) was added to the EVT differentiation media on Day 1 (2% v/v Cultrex) and Day 3 (0.5% v/v Cultrex). Neuregulin (100 ng/ml; 26941S, Cell Signaling Technology, Danvers, MA, USA) was added to the EVT differentiation media on Day 1 only.

### 3D matrix-free cultures via cell aggregation

AggreWell 800 culture plates (34815, 24-well plates or 34825, 6-well plates; STEMCELL Technologies) were pretreated with an anti-adherence rinsing solution (07010, STEMCELL Technologies) and centrifuged at 1300×*g* for 5 min. After inspecting under a microscope to ensure the absence of bubbles, the plates were rinsed with prewarmed basal TS media. Human TS or HTR cells (9.0 × 10^5^ cells per 24-well or 4.5 × 10^6^ cells per 6-well; 3000 cells per microwell) were resuspended in complete TS media, whereas BeWo cells were added at the same concentration in BeWo media. The plates were centrifuged at 100×*g* for 3 min and incubated in a humidified incubator at 37°C and 5% CO_2_. After 48 h, the resulting 3D cultures were dislodged with basal media, collected with a 37-µm reversible strainer (27215, STEMCELL Technologies), and transferred into ultra-low attachment 6-well plates (3471, Corning Inc.). The 3D cultures were grown for four more days in complete TS media and monitored with a phase-contrast microscope (Leica Microsystems, Wetzlar, Germany). At this point, 3D cultures were collected for RNA or protein isolation. Alternatively, the 3D cultures underwent fixation with 4% paraformaldehyde for 1 h, followed by overnight incubation in 30% sucrose, embedding in optimal cutting temperature media (Tissue-Tek, Torrance, CA, USA), and storage at −80°C prior to cryosectioning. The 3D cultures were sectioned on a Cryostar NX50 cryostat (Thermo Fisher Scientific) at 10 µm thickness.

### Small molecule inhibitors

For small molecule inhibitor experiments, 3D cultures were treated with 10 μM LATS-IN-1 (36623, Cayman Chemicals, Burlington, ON, Canada), an inhibitor of the Hippo pathway also called TRULI (Kastan *et al.*, 2021), or an equivalent volume of dimethyl sulfoxide (DMSO) in complete TS media for 6 days. To quantify the area of 3D cultures, five fields of view were selected per well and imaged with a phase-contrast microscope. These images were transformed into binary images, eroded five times, and the resulting particles analyzed with Fiji (ImageJ version 1.54 g) (Schindelin *et al.*, 2012).

### Quantitative RT-PCR

Per the manufacturer’s instructions, RNA was isolated from 2D and 3D cultures with TRIzol (Thermo Fisher Scientific) or TRAzol (GeneBio Systems, Burlington, ON, Canada). Purified RNA was converted through reverse transcription into complementary DNA (cDNA; High-Capacity cDNA Reverse Transcription Kit, Thermo Fisher Scientific). After diluting cDNA 1:10, GB-Amp Sybr Green qPCR Mix (GeneBio Systems) and forward and reverse primers (Supplementary Table S1) were added for quantitative RT-PCR. A CFX96 Connect Real-Time PCR Detection System (Bio-Rad Laboratories, Mississauga, ON, Canada) was used for amplification and fluorescence detection. Cycling conditions involved an initial holding step (95°C for 10 min), 40 cycles of 2-step PCR (95°C for 15 s, followed by 60°C for 1 min), and concluded with a dissociation phase. Relative mRNA levels were calculated through the comparative cycle threshold (2^-ΔΔCt^) method (Livak and Schmittgen, 2001), using the geometric means of Ct values of two reference RNAs, *EEF2* and *GAPDH* (for BeWo cells) or *EEF2* and *YWHAZ* (all other cells), for normalization.

### Immunofluorescence

Cryosections and 2D cells were fixed in 4% paraformaldehyde, permeabilized with 0.3% Triton X-100, and blocked with 5% goat serum (Thermo Fisher Scientific). For YAP1 staining, samples first underwent antigen retrieval by submersion in 1 mM ethylenediaminetetraacetic acid, pH 8.0 containing 0.05% Tween-20 at 95°C for 30 min, followed by cooling to room temperature before blocking. Samples were then incubated with primary antibodies specific for chorionic gonadotropin beta (CGB; 1:500, PA5-16265, Thermo Fisher Scientific), E-cadherin (CDH1; 1:200, 3195P, Cell Signaling Technology), hCG (1:400, ab9376, Abcam, Cambridge, UK), human leukocyte antigen G (HLA-G; 1:200, 21799, Santa Cruz Biotechnology, Dallas, TX, USA), the proliferation marker Ki67 (1:100, ab15580, Abcam), TEAD4 (1:100, HPA056896, Sigma-Aldrich), YAP1 (1:100, ab205270, Abcam), and zonula occludens-1 (ZO-1; 1:100, 33-9100, Thermo Fisher Scientific). Afterwards, samples were incubated with species-specific Alexa Fluor 488 or Alexa Fluor 555-conjugated antibodies, nuclei counterstained with 4′,6-diamidino-2-phenylindole (DAPI, Thermo Fisher Scientific), and mounted with Fluoromount-G mounting medium (Thermo Fisher Scientific). Images were acquired using a Nikon DS-Qi2 microscope (Nikon Instruments Inc., Melville, NY, USA) or a Leica MateoFL system (Leica Microsystems). ImageJ v1.54 was used for quantitative analysis of fluorescence intensities and positive signal areas. For hCG, quantification was performed by calculating the ratio of hCG-positive area to cell number in each field of view. For Ki67 and TEAD4, quantification was performed by calculating the ratio of positive nuclei to total nuclei in each field of view. In all cases, data were normalized to the control groups: either 2D or DMSO-treated cells, as appropriate. For the percentage of cytoplasmic YAP1, total fluorescence intensity was first measured. Nuclear YAP1 intensity was then quantified by using the DAPI image to define the size and position of nuclei and applying this nuclear mask to the corresponding YAP1 image. Cytoplasmic YAP1 intensity was calculated by subtracting nuclear YAP1 intensity from total YAP1 intensity and expressing this value as a percentage of total YAP1 intensity.

### Western blotting

2D and 3D culture lysates were incubated with radioimmunoprecipitation buffer (150 mM NaCl, 50 mM Tris, 1% NP-40, 0.5% sodium deoxycholate, 0.1% sodium dodecyl sulfate (SDS)) and protease inhibitor cocktail (P8340, Sigma-Aldrich). Protein concentration was quantified through a bicinchoninic acid assay, which was performed according to the manufacturer’s instructions (Bio-Rad Laboratories). The protein samples were mixed with a 4× reducing loading buffer (0.25 M Tris, 8% SDS, 0.02% bromophenol blue, 0.3 M dithiothreitol), heated at 95°C for 5 min, and loaded into an SDS-containing polyacrylamide gel. After electrophoresis, the proteins were transferred onto a polyvinylidene difluoride membrane, which was incubated with primary antibodies specific for hCG (1:5000, ab9376), YAP1 (1:1000, 14074, Cell Signaling Technology), phosphorylated (Ser127) YAP1 (p-YAP1; 1:1000, 13008, Cell Signaling Technology), and ACTB (1:1000, sc-47778, Santa Cruz Biotechnology). Afterwards, the membranes were incubated with species-appropriate secondary antibodies. The samples were examined with a LI-COR Odyssey imaging system (LI-COR, Lincoln, NE, USA), and band intensity relative to ACTB was quantified using Image Lab v6.0 (Bio-Rad Laboratories).

### ELISA

The levels of secreted CGB were measured with an ELISA kit, following instructions from the manufacturer (EIA1911R, DRG International, Springfield, NJ, USA). Media were collected from 2D and 3D human TS cultures, cellular debris was removed through centrifugation, and the supernatant was diluted with the provided sample diluent. Afterwards, the diluted supernatants were added to a microplate precoated with a CGB antibody alongside an enzyme conjugate. Following washing steps, a colorimetric reaction was started through addition of a substrate solution containing tetramethylbenzidine and halted with a stop solution containing 0.5 M sulfuric acid. From the absorbance measurements, a standard curve was created based on standards of known concentration, which enabled interpolation of the sample concentration values. The sensitivity of this ELISA was 0.44 mIU/ml.

### RNA-sequencing

For RNA isolation, 2D and 3D human TS cultures were lysed with TRAzol. The aqueous layer was extracted and diluted with 70% ethanol. RNA was purified through the RNeasy mini kit (74104, Qiagen, Toronto, ON, Canada), and DNase I (79254, Qiagen) was added to remove contaminant DNA. RNA samples were sent to Génome Québec (Montréal, QC, Canada), with RNA integrity evaluated with a Bioanalyzer (Agilent Technologies, Mississauga, ON, Canada) and libraries constructed with a NEBNEXT Ultra RNA Library Prep Kit for Illumina (New England Biolabs, Whitby, ON, Canada). All samples achieved an RNA integrity number ≥ 8.6. Sequencing of libraries was performed on a NovaSeq 6000 (Illumina, San Diego, CA, USA), with 100 bp paired-end reads for approximately 25 million reads per sample. One sample was run separately due to an initial library generation failure that was resolved on a subsequent attempt. The quality of libraries or sequencing did not differ between batches.

Analyses were generated through Partek Flow (Illumina). Quality control was performed on the fastq files, which were imported and assigned attributes based on type (2D, 3D) and replicate. Reads were aligned to the human reference genome (GRCh38) with STAR (v2.7.8.a) and quantified for annotation with Ensembl Transcripts Release 110. Genes with fewer than 12 reads were filtered out (24 139 genes reduced to 19 592). Counts were normalized with mean ratio, and a differential gene expression analysis (DESeq2) was generated for 18 996 genes. Genes with a false discovery rate (FDR) value < 0.01 and an absolute fold change ≥ 2 between 2D and 3D human TS cultures were considered significantly different. Gene Ontology (GO) and Kyoto Encyclopedia of Genes and Genomes (KEGG) pathway enrichment analyses (Ashburner *et al.*, 2000; Kanehisa and Goto, 2000) were performed on these sets of significantly different downregulated and upregulated genes through Partek Flow and visualized with SRplot (Tang *et al.*, 2023).

### YAP1/TAZ-TEAD reporter assay

Human TS cells (1.25 × 10^4^) were plated onto collagen-coated 24-well plates. The next day, 200 ng of vector containing a YAP1/TAZ-responsive synthetic promoter consisting of eight copies of the GTIIC motif driving firefly luciferase expression (34615, Addgene, Watertown, MA, USA) was transfected into 2D human TS cultures with jetPRIME transfection reagent (Polyplus Transfection, New York, NY, USA). The plasmid was a gift from Stefano Piccolo (Dupont *et al.*, 2011). After 18 h of transfection, human TS cultures were treated with 10 μM of LATS-IN-1 or an equivalent volume of DMSO in complete TS media for 24 h. Cells were lysed with the Bright-Glo Luciferase assay system (E2610, Promega, Madison, WI, USA), with luciferase activity measured using a VICTOR^3^V luminescence microplate reader (PerkinElmer, Waltham, MA, USA).

### Statistical analysis

Analyses of RNA-sequencing data and immunofluorescence images were described above. For all other analyses, statistical comparisons involving two means were evaluated using a Student’s *t*-test with two tails. One-way analysis of variance was performed with Tukey’s post-hoc test for multiple comparisons between three or more means. All graphing and statistical analyses were conducted on GraphPad Prism 8 or 10 (San Diego, CA, USA). Each experiment was repeated at least three independent times. *P *< 0.05 was used as the threshold for statistical significance.

## Results

### Validation of the stem and differentiation capacities of human TS cells

2D human TS cultures were maintained in stem conditions or cultured in STB or EVT differentiation media to confirm their capacity to form these subpopulations. Under stem conditions, human TS cells retained a colony-reminiscent morphology. Expression of the CTB marker, *TEAD4*, was significantly decreased by 5.5- and 4.5-fold in human TS cells under STB and EVT differentiation conditions, respectively (Supplementary Fig. S1, *P *< 0.0001). Culturing human TS cells in STB differentiation media resulted in fusion and increased expression of the STB marker, *CGB*, by 47-fold (Supplementary Fig. S1, *P *< 0.001). In EVT differentiation conditions, human TS cells developed a spiky morphology, and expression of EVT markers, *HLA-G* and *NOTUM*, was elevated by 3- and 62-fold, respectively (Supplementary Fig. S1, *P *< 0.05). Altogether, these results substantiate that human TS cells can form STB and EVT when cultured under the appropriate conditions.

### Human TS cells cultured in 3D spontaneously undergo STB differentiation

Next, we examined whether 3D matrix-free culture was associated with changes in trophoblast subpopulation identity in human TS cells. Based on our previous protocol (Jeyarajah *et al.*, 2022), 3D human TS cultures were aggregated in AggreWell plates for 48 h and cultured in suspension for an additional four days in complete TS media (Fig. 1a). Afterwards, 3D human TS cultures were compared with 2D cultures maintained in the same media composition.

**Figure 1.**
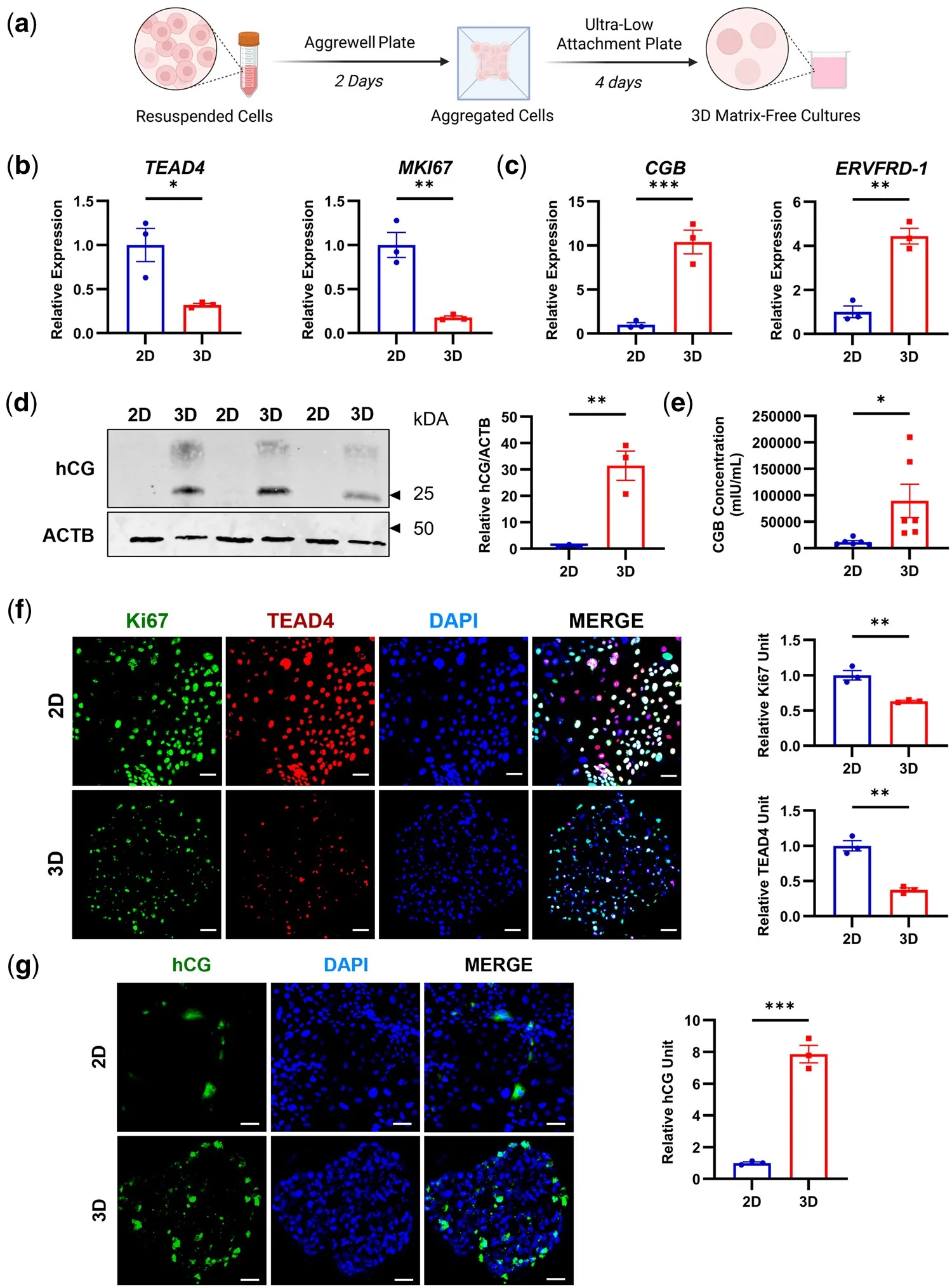
**Increased syncytiotrophoblast differentiation in three-dimensional matrix-free human trophoblast stem cultures**. Two-dimensional (2D) and three-dimensional (3D) human trophoblast stem (TS) cultures were grown in complete TS media. (**a**) Schematic detailing how 3D trophoblast cultures were generated. Created in BioRender. Zheng, M. (2025) https://BioRender.com/9kcgv03. (**b** and **c**) Quantitative RT-PCR for (b) cytotrophoblast (CTB; *TEAD4*, *MKI67*) and (c) syncytiotrophoblast (STB; *CGB*, *ERVFRD-1*) markers; n = 3 independent experiments. (**d**) Western blot for the STB marker hCG, with ACTB serving as the loading control (left panel). A graph showing relative band intensity is shown on the right; n = 3 independent experiments. (**e**) CGB levels in conditioned media; n = 6 independent experiments. (**f** and **g**) Immunofluorescence for (f) Ki67 and TEAD4 and (g) hCG. Nuclei were stained with DAPI in both panels. Quantification of the relative number of cells that were positive for these markers is shown on the right; n = 3 independent experiments. The scale bars equal 50 µm. Graphs show mean ± SEM. Student’s *t*-test. *P < 0.05, **P < 0.01, ***P < 0.001.

Expression of CTB markers, *TEAD4* and *ITGA6*, was downregulated in 3D compared to 2D cultures (Fig. 1b and Supplementary Fig. S2a, *P *< 0.05). Expression of *MKI67*, which encodes the proliferation-associated marker Ki67, decreased by 6-fold in 3D human TS cultures compared to their 2D counterparts (Fig. 1b, *P *< 0.01). Interestingly, transcript levels of *CGB* and *CGA*, which encode subunits of the characteristic STB hormone hCG, both increased by approximately 10-fold in 3D compared to 2D human TS cultures (Fig. 1c and Supplementary Fig. S2b, *P *< 0.01). The more modest upregulation of *CGB* under this protocol, compared to 2D STB differentiation (Supplementary Fig. S1), likely reflects spontaneous differentiation of a subset of cells in 3D cultures rather than robust differentiation driven by removal of stem media components and addition of forskolin. Similarly, the endogenous retroviral genes *ERVFRD-1* and *ERVW-1*, which encode proteins implicated in trophoblast fusion during STB formation, were also upregulated in 3D cultures (Fig. 1c and Supplementary Fig. S2b, *P *< 0.01). At the protein level, 3D human TS cultures showed a substantial increase in hCG in cell lysates and CGB in conditioned media compared to 2D cultures, as determined through western blotting (Fig. 1d, *P *< 0.01) and ELISA (Fig. 1e, *P *< 0.05), respectively. Uncropped western blots are shown in Supplementary Fig. S3.

The CTB marker TEAD4 and proliferation marker Ki67 were readily detectable in 2D human TS cultures, with most (but not all) nuclei positive for both proteins. In contrast, nuclei appeared more compacted in 3D cultures, and TEAD4 and Ki67 were detected in relatively fewer nuclei (Fig. 1f, *P *< 0.01). Conversely, while CGB was detectable in few cells in 2D cultures, it was enriched in 3D human TS cultures, particularly at the periphery in multinucleated aggregates, consistent with outward STB differentiation (Fig. 1g, *P *< 0.001 and Supplementary Fig. S2c). Overall, these findings indicate that human TS cultures undergo spontaneous STB differentiation when cultured in 3D.

### BeWo cells exhibit increased hCG levels in 3D culture, consistent with an STB-like response

Subsequently, we investigated whether 3D matrix-free culturing would induce a similar transformation in trophoblast subpopulations with the widely-used BeWo and HTR trophoblast cell lines. The 2D and 3D HTR cultures underwent the same protocol in complete TS media as the human TS cells, while BeWo cells were kept in their original media conditions.

In 3D BeWo cultures, there appeared to be reduced *TEAD4* expression compared to 2D, although this was variable and did not reach statistical significance (Fig. 2a). However, *CGB* was upregulated 12-fold in 3D compared to 2D cultures (Fig. 2b, *P *< 0.05). Similarly, expression of other STB markers, *CGA* and *ERVFRD-1*, increased by 12- and 3-fold when BeWo cells were cultured in 3D, respectively (Fig. 2b, *P *< 0.05). Immunofluorescence also showed a significant increase in hCG in BeWo cells cultured in 3D (Fig. 2c, *P *< 0.01).

**Figure 2.**
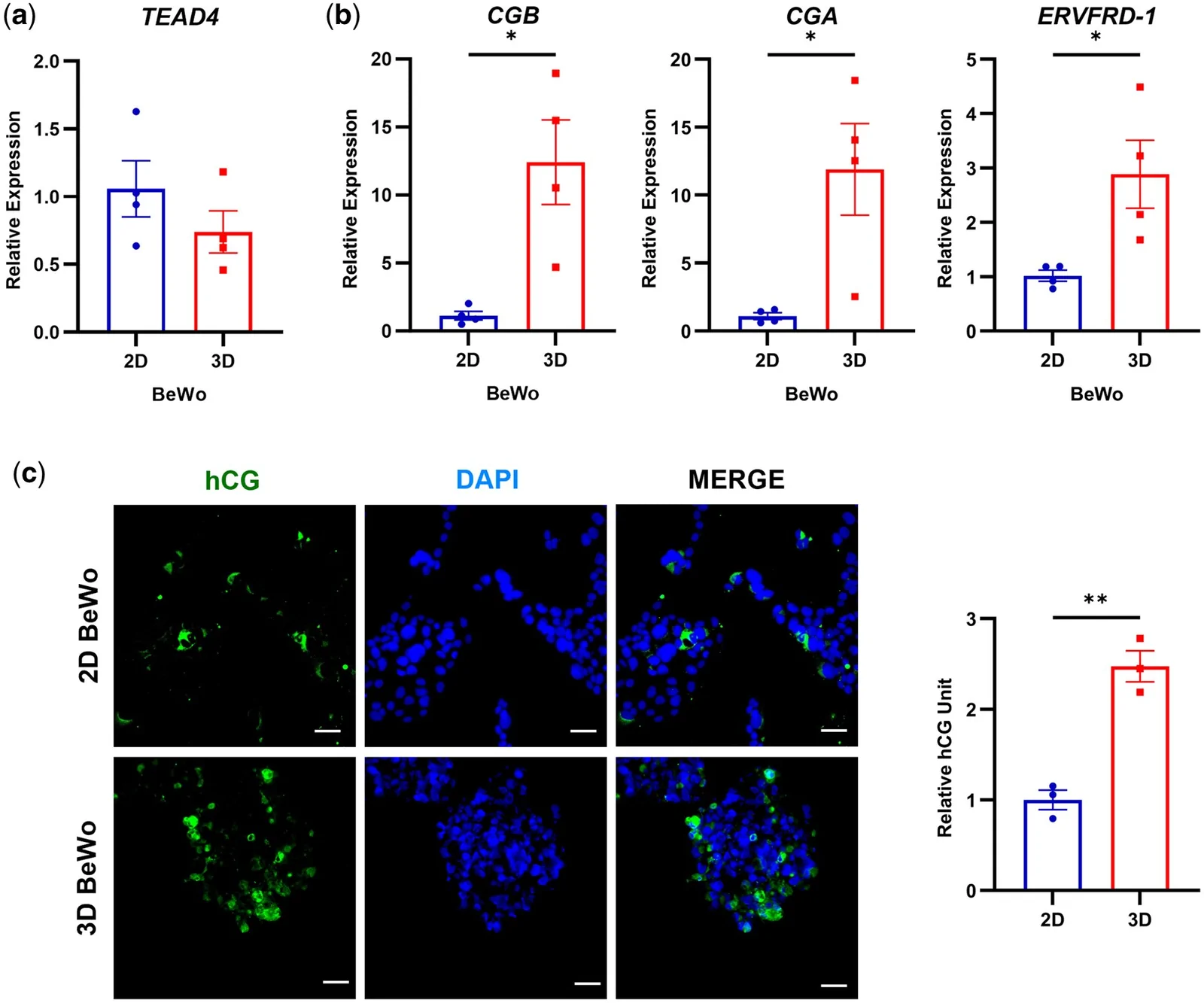
**Three-dimensional culture promotes syncytiotrophoblast-associated features in BeWo cells**. (**a** and **b**) Quantitative RT-PCR for (a) cytotrophoblast (CTB; *TEAD4*) and (b) syncytiotrophoblast (STB; *CGB*, *CGA*, *ERVFRD-1*) markers between two-dimensional (2D) and three-dimensional (3D) BeWo cultures; n = 4 independent experiments. (**c**) Immunofluorescence for hCG in 2D and 3D BeWo cultures. Nuclei were stained with DAPI. The relative number of cells that were positive for hCG is shown on the right; n = 3 independent experiments. The scale bars equal 50 µm. Graphs show mean ± SEM. Student’s *t*-test. *P < 0.05, **P < 0.01.

In HTR cells, *TEAD4* was highly expressed in 2D cultures, and its expression was downregulated 1.6-fold when cells were cultured in 3D (Supplementary Fig. S4a, *P *< 0.05). However, based on their Ct values, the CTB marker *ITGA6* and several STB markers, including *CGB* and *ERVW-1*, were expressed at low levels in these cells. Within this low-expression range, 3D culture did not alter expression of any of these genes (Supplementary Fig. S4a and b). Consistent with this observation, although *TEAD4* transcript levels were reduced in 3D culture, TEAD4 protein localization remained readily detectable, whereas CGB was not detected by immunofluorescence in 3D HTR culture (Supplementary Fig. S4c and d). Collectively, these findings suggest that BeWo cells, but not HTR cells, exhibit features consistent with a partial STB-like response when cultured in 3D.

### 3D culture increases expression of genes associated with differentiated trophoblasts in human TS cells

From the previous findings, we established that human TS cells undergo spontaneous syncytialization in 3D culture, while BeWo cells exhibit a partial STB-like response under similar conditions. As such, we investigated how the transcriptome shifts between 2D and 3D human TS cultures through bulk RNA-sequencing. Principal component analysis revealed that 85.93% of the variability was accounted for by PC1, which separates 2D and 3D human TS cultures. While replicates for both groups cluster closely, there was more variability among the 3D human TS cultures (Fig. 3a). Out of the 18 996 transcripts annotated, there was significant (FDR < 0.01) upregulation of 2091 genes and downregulation of 1433 genes above 2-fold between 3D and 2D human TS cultures (Fig. 3b and Supplementary Table S2). These findings suggest that the transcriptomes of 3D human TS cultures differ markedly from those of their 2D culture counterparts despite being exposed to the same media composition.

**Figure 3.**
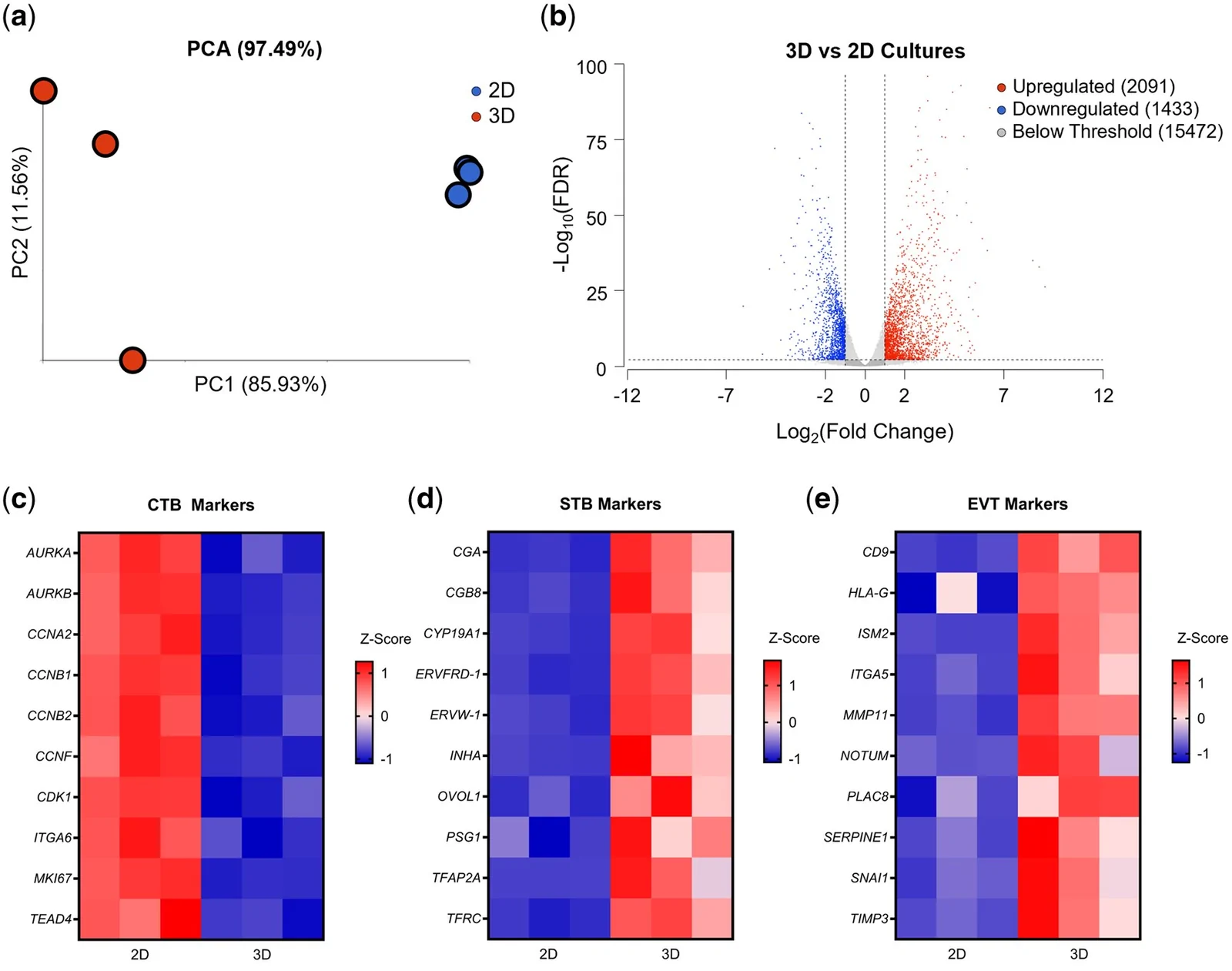
**Global gene expression profiling of three-dimensional human trophoblast stem cultures**. Two-dimensional (2D) and three-dimensional (3D) human trophoblast stem (TS) cultures grown in complete TS media were collected for RNA sequencing; n = 3 independent experiments. (**a**) Principal component analysis (PCA) plot. (**b**) Volcano plot, with genes significantly (false detection rate, FDR < 0.01) upregulated (red) and downregulated (blue) over 2-fold between 3D and 2D cultures. (**c–e**) Heatmaps for (c) cytotrophoblast (CTB), (d) syncytiotrophoblast (STB), and (e) extravillous trophoblast (EVT) markers.

An extended list of trophoblast subpopulation markers based on human TS (Okae *et al.*, 2018; Saha *et al.*, 2020; Varberg *et al.*, 2023) and 3D trophoblast organoid (Haider *et al.*, 2018, 2022; Sheridan *et al.*, 2021; Shannon *et al.*, 2024) studies was examined with these differentially regulated genes. For expression of CTB markers, there was a significant downregulation of *TEAD4* and *ITGA6* by 2-fold in 3D human TS cultures (Fig. 3c). Expression of many cell cycle regulators (*CCNA2*, *CCNB1*, *CCNB2*, *CCNF*, and *CDK1*) was also decreased (Fig. 3c), consistent with the notion that CTB self-renewal is reduced when human TS cells are cultured in 3D. Other notably downregulated CTB markers in 3D human TS cultures include genes encoding microtubule-associated proteins, *AURKA* and *AURKB* (3- and 4-fold), and the proliferation marker, *MKI67* (6-fold; Fig. 3c).

Conversely, *CGA* and *CGB* transcripts (*CGB1*, *CGB2*, *CGB3*, *CGB5*, *CGB7*, and *CGB8*) were significantly increased by 8- to 11-fold in 3D human TS cultures compared with 2D cultures (Fig. 3d). Other STB endocrine-related markers, including *CYP19A1*, *INHA*, and *PSG1*, were also upregulated by 10-, 12-, and 3-fold, respectively (Fig. 3d). Similarly, STB differentiation and fusion-associated markers, including *OVOL1*, *TFAP2A*, *ERVW-1*, and *ERVFRD-1*, were upregulated in 3D cultures, as was *TFRC*, a marker associated with STB microvilli (Fig. 3d).

Intriguingly, levels of several EVT-associated markers involved in cell invasion and extracellular matrix remodelling were increased in 3D human TS cultures compared with 2D cultures, including *CD9*, *MMP11*, *PLAC8*, *SERPINE1*, and *TIMP3* (Fig. 3e). *SNAI1*, which encodes a transcriptional regulator associated with EVT differentiation, was also upregulated in 3D cultures, as were levels of other EVT-associated genes, including *ISM2*, *ITGA5*, and *NOTUM* (Fig. 3e). Expression of the prototypical EVT marker *HLA-G* appeared to be upregulated in 3D cultures but fell just below the 2-fold threshold used for this analysis (1.96-fold increase; Fig. 3e). To determine whether HLA-G was detectable at the protein level, we performed immunofluorescence. Scattered HLA-G-positive cells were detected throughout the cultures, although staining was variable and did not exhibit the same consistent pattern or intensity observed for STB markers (Supplementary Fig. S2d). Thus, while increased expression of EVT-associated genes in 3D cultures was noteworthy, it was less pronounced than the induction of genes linked with STB differentiation and function. Together, these findings suggest that 3D matrix-free culture reduces CTB-associated gene expression while promoting transcriptional changes associated with spontaneous differentiation, with STB differentiation appearing more prominent than EVT differentiation under these conditions.

### Altered cell cycle and YAP1/TAZ-TEAD signaling in 3D human TS cultures

Subsequently, we performed gene set enrichment analysis to identify putative pathways and cellular functions altered between 2D and 3D human TS cultures. Downregulated and upregulated genes were analyzed separately, and potential signaling pathways associated with 3D culture were identified from KEGG environmental information processing pathways. Hippo and Wnt-related signaling were among the most significant for the 3D-downregulated genes (Fig. 4a), both of which are known to maintain the CTB stem state (Pollheimer *et al.*, 2006; Okae *et al.*, 2018; Saha *et al.*, 2020). Among the 3D-upregulated genes, the most significant KEGG environmental information processing pathway was hypoxia inducible factor-1 (HIF-1; Fig. 4a), which may indicate hypoxia or metabolic stress resulting from the 3D arrangement of cells.

**Figure 4.**
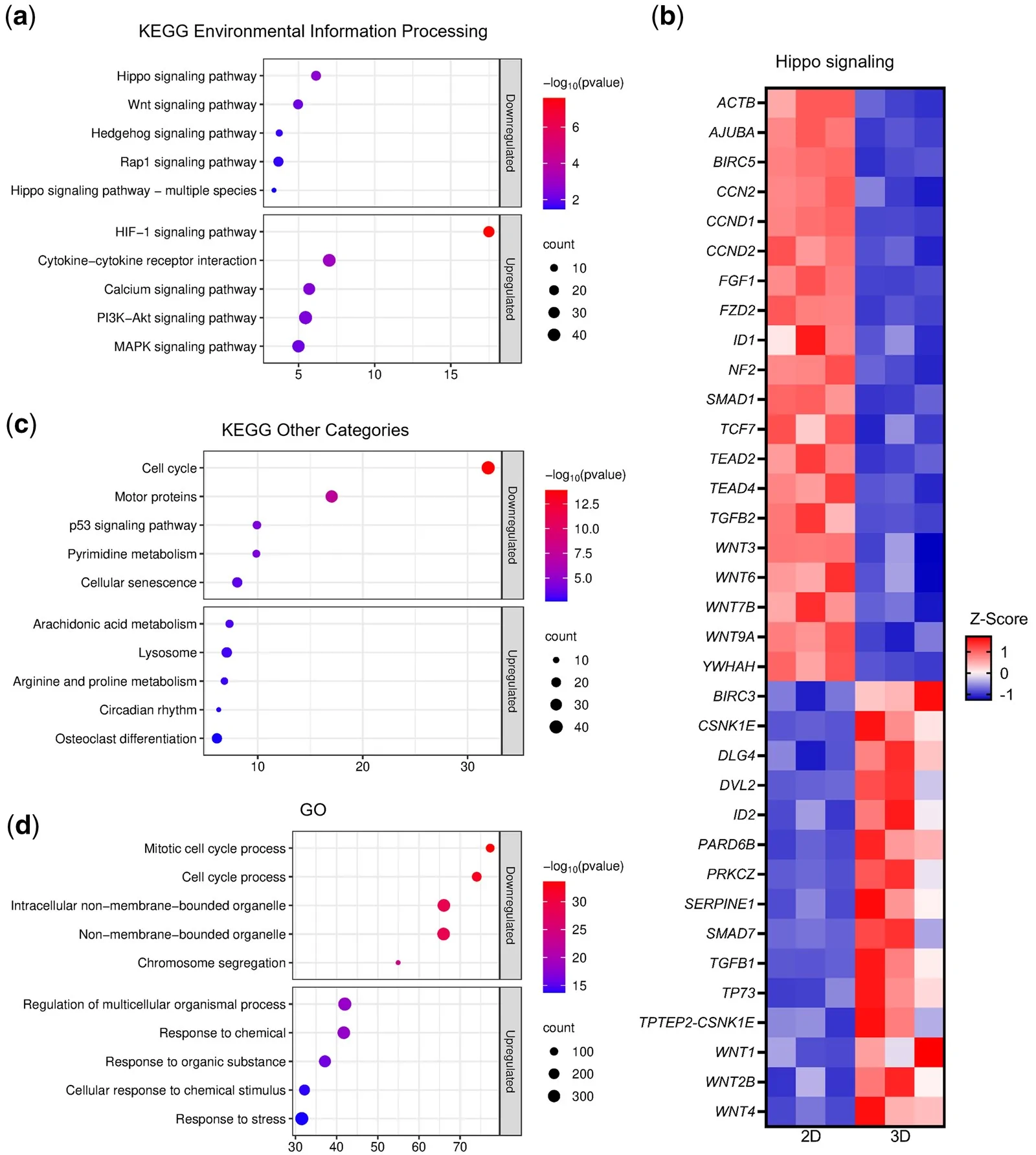
**Predicted pathways modified by three-dimensional matrix-free culturing with human trophoblast stem cells**. Two-dimensional (2D) and three-dimensional (3D) human trophoblast stem (TS) cultures grown in complete TS media were processed for RNA-sequencing; n = 3 independent experiments. (**a**) The five most significant Kyoto Encyclopedia of Genes and Genomes (KEGG) environmental information processing pathways downregulated and upregulated in 3D cultures. (**b**) Heatmap for differentially regulated targets associated with the Hippo pathway. (**c** and **d**) The five most significant (c) KEGG pathways from other categories and (d) gene ontology (GO) pathways downregulated and upregulated in 3D cultures.

To further explore the potential involvement of Hippo signaling, we examined genes within the ‘Hippo pathway’ term that were differentially expressed between 2D and 3D human TS cultures. Of the 157 genes included in this pathway, 35 were differentially expressed between culture conditions. Several genes associated with YAP1/TAZ-TEAD transcriptional activity were downregulated in 3D cultures, including *TEAD2* and *TEAD4*, while expression of multiple YAP1/TAZ-associated target genes involved in proliferation, survival, and anti-apoptotic signaling were also altered, including *NF2*, *YWHAH*, *BIRC3*, *BIRC5*, *CCN2*, *CCND1*, *CCND2*, *FGF1*, *ID1, ID2*, and *SERPINE1* (Fig. 4b). Genes that intersect with Wnt and TGFβ signaling were also differentially expressed between culture conditions, including *DVL2*, *FZD2*, multiple WNT ligands, *SMAD1*, *SMAD7*, *TGFB1*, and *TGFB2* (Fig. 4b). Together, these findings suggest that 3D culture is associated with altered expression of genes associated with the Hippo pathway, including changes in core TEAD transcription factors and downstream YAP1/TAZ-associated transcriptional programs.

From the genes downregulated in 3D cultures, the most significant KEGG pathways from the other categories were related to the cell cycle and cytoskeleton (Fig. 4c), which are likely associated with reduced cell proliferation and increased differentiation when cultured in 3D. Likewise, the GO analysis of the 3D-downregulated genes identified similar biological processes involving the cell cycle, chromosomes, and microtubules (Fig. 4d). Additionally, the 3D-downregulated genes yielded KEGG pathways associated with transcription and nucleotide metabolism (Fig. 4c). Overall, these findings suggest a decreased presence of mitotically-active CTB in 3D cultures.

Among the 3D-upregulated genes, the top enriched KEGG pathways included autophagy and lipid metabolism (Fig. 4c). GO analysis further revealed enrichment of biological processes associated with regulation of multicellular processes, cellular activity, and responses to chemical, environmental, and stress-related stimuli, which may reflect adaptation to 3D organization (Fig. 4d). Collectively, this functional analysis revealed reduced expression of genes associated with cell cycle progression in 3D human TS cultures, alongside pathway-level changes suggesting potential involvement of Hippo signaling.

### LATS1/2 inhibition reduces spontaneous STB formation in 3D human TS cultures

Next, we investigated whether Hippo/YAP1 pathway activity contributes to spontaneous STB differentiation by treating 3D human TS cultures with LATS-IN-1, which inhibits LATS1/2-mediated phosphorylation of YAP1/TAZ and thereby increases YAP1/TAZ-TEAD transcriptional activity (Kastan *et al.*, 2021). Previous studies have used LATS-IN-1 at concentrations of 5–10 µM to inhibit Hippo signaling in several 3D cell models, including retinal organoids (Kastan *et al.*, 2021), blastoids (Yu *et al.*, 2023), breast cancer spheroids (Ramírez-Cuéllar *et al.*, 2024), and embryos (Gerri *et al.*, 2023). Based on these studies, 3D human TS cultures were treated with 10 µM LATS-IN-1, while controls were treated with an equivalent volume of DMSO.

To confirm that LATS-IN-1 induced YAP1/TAZ-TEAD transcriptional activity, 2D human TS cells were transfected with a vector encoding firefly luciferase under the control of a YAP1/TAZ-responsive synthetic promoter. Luminescence measurements showed that TEAD transcriptional activity was approximately 3-fold higher in TS cells treated with LATS-IN-1 compared with DMSO controls (Supplementary Fig. S5a, *P *< 0.05). In 3D cultures, LATS-IN-1 treatment also reduced YAP1 phosphorylation (Fig. 5a, *P *< 0.001) and reduced cytoplasmic localization of YAP1 (Fig. 5b, *P *< 0.01), consistent with inhibition of LATS1/2 activity and activation of YAP1/TAZ-TEAD signaling.

**Figure 5.**
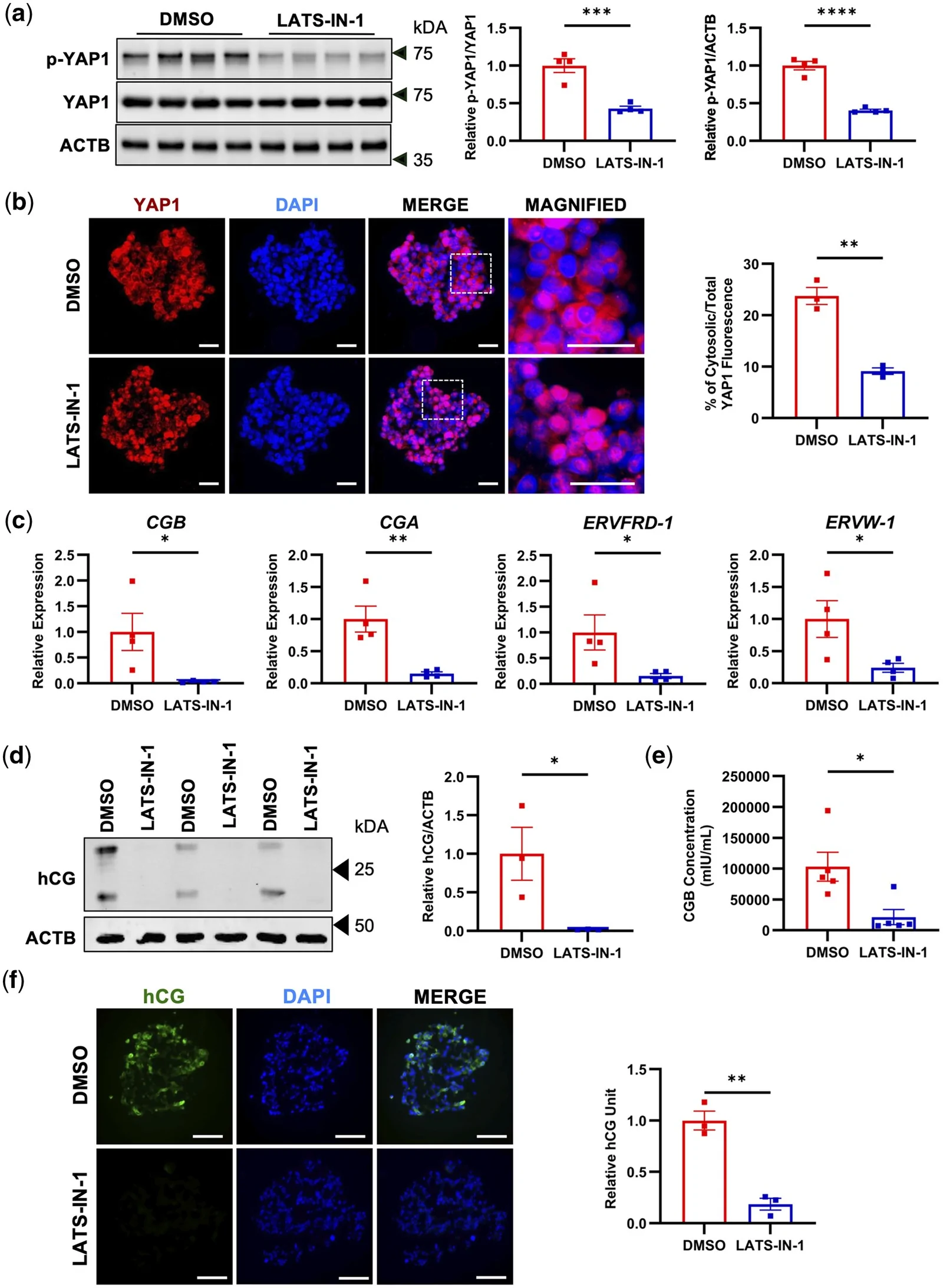
**Inhibition of LATS1/2 diminishes syncytiotrophoblast formation in three-dimensional human trophoblast stem cultures**. (**a**) Western blot showing phosphorylated YAP1 (p-YAP1) and total YAP1 in three-dimensional (3D) human trophoblast stem (TS) cultures exposed to DMSO (vehicle) or 10 μM LATS-IN-1. Quantification of p-YAP1 band intensity relative to total YAP1 and ACTB is shown to the right; n = 4 independent experiments. (**b**) Immunofluorescence for YAP1 in 3D human TS cultures exposed to DMSO or LATS-IN-1. Nuclei were stained with DAPI. The boxed region in the merged image indicates the area shown at higher magnification. Quantification of cytoplasmic YAP1 signal is shown to the right of the representative images; n = 3 independent experiments. (**c**) Quantitative RT-PCR analysis of syncytiotrophoblast (STB)-associated markers (*CGB*, *CGA, ERVFRD-1*, and *ERVW-1*); n = 4 independent experiments. (**d**) Western blot for hCG, with ACTB as the loading control. A graph showing relative hCG band intensity normalized to ACTB is shown to the right; n = 3 independent experiments. (**e**) CGB levels in media; n = 5 independent experiments. (**f**) Immunofluorescence for hCG (green), with nuclei stained using DAPI (blue). The relative number of cells that were positive for hCG is shown on the right; n = 3 independent experiments. The scale bars equal 50 µm. Graphs show mean ± SEM. Student’s *t*-test. *P < 0.05, **P < 0.01, ***P < 0.001, ****P < 0.0001.

Treatment of 3D human TS cultures with LATS-IN-1 appeared to reduce the peripheral ‘bubble-like’ morphology observed in DMSO-treated controls, suggesting reduced spontaneous STB formation at the periphery (Supplementary Fig. S5b). In addition, LATS-IN-1 treatment showed a trend toward increased mean surface area across three independent experiments, although the difference did not reach statistical significance (Supplementary Fig. S5c, *P *= 0.06). Immunofluorescence further showed an increased proportion of Ki67-positive cells following LATS-IN-1 treatment (Supplementary Fig. S5d, *P *< 0.01), consistent with enhanced proliferation.

Expression of the CTB markers *TEAD4* and *ITGA6* did not differ between 3D human TS cultures treated with DMSO or LATS-IN-1 (Supplementary Fig. S5e). Nevertheless, LATS-IN-1 treatment substantially decreased expression of endocrine-associated STB markers, including *CGB* by 31-fold (Fig. 5c, *P *< 0.05), *CGA* by 7-fold (Fig. 5c, *P *< 0.01), and *HSD11B2* by 1.8-fold (Supplementary Fig. S5f, *P *< 0.05). Similarly, STB markers involved in fusion, including *ERVFRD-1* (Fig. 5c, *P *< 0.05) and *ERVW-1* (Fig. 5c, *P *< 0.05), were downregulated by 7- and 4-fold following LATS-IN-1 treatment.

LATS-IN-1 treatment decreased hCG protein levels by 57-fold in lysates (Fig. 5d, *P *< 0.05) and secreted CGB levels by 5-fold in conditioned media (Fig. 5e, *P *< 0.05) from 3D human TS cultures compared with DMSO controls. Similarly, hCG immunofluorescence, which was enriched along the periphery of DMSO-treated 3D human TS cultures, was markedly reduced following LATS-IN-1 treatment (Fig. 5f, *P *< 0.01). In sum, these findings indicate that LATS1/2 inhibition, which activates YAP1/TAZ-TEAD signaling, reduces spontaneous STB differentiation in 3D human TS cultures.

One possible explanation for the reduced presence of STB in 3D human TS cultures treated with LATS-IN-1 is that cells may instead be directed toward the EVT lineage. However, *HLA-G* expression did not differ between groups (Supplementary Fig. S5g). Although the EVT marker *NOTUM* was upregulated approximately 2-fold in LATS-IN-1-treated 3D cultures (Supplementary Fig. S5g, *P *< 0.001), expression of another EVT-associated marker, *ITGA5*, was decreased approximately 1.5-fold (Supplementary Fig. S5g, *P *< 0.05). Notably, all three EVT-associated markers were expressed at relatively low levels overall, based on high Ct values, compared with genes associated with CTB and STB. Together, these findings do not support enhanced EVT differentiation as the primary explanation for reduced STB formation in LATS-IN-1-treated 3D human TS cultures.

## Discussion

The placenta plays a central role in shaping fetal and maternal outcomes, underscoring the need for more representative *in vitro* models. While culture systems that better represent the 3D organization of the human placenta have been developed, the mechanisms regulating trophoblast development within these models remains unclear. In this study, we identified changes in trophoblast subpopulations within 3D matrix-free trophoblast cultures and examined the role of Hippo pathway-associated signaling in regulating spontaneous differentiation. 3D human TS cultures exhibited reduced CTB marker expression accompanied by enhanced spontaneous differentiation, with STB differentiation representing the predominant trajectory. This outward STB formation establishes a more physiologically relevant organization of trophoblast subpopulations. KEGG analysis of genes downregulated in 3D human TS cultures revealed potential involvement of Hippo signaling, and functional experiments with LATS-IN-1 suggest that reduced YAP1/TAZ-TEAD activity contribute to spontaneous STB formation.

Culturing human TS cells in a 3D, matrix-free environment reduces the proportion of CTBs despite being cultured in stem-promoting media. Specifically, several characteristic CTB markers were downregulated, such as those regulating the cell cycle and transcription factors that maintain stemness, in 3D matrix-free human TS cultures. The prevalence of cell cycle-related terms associated with the 3D-downregulated genes further indicates a reduced proportion of mitotically-active CTBs. This transformation in 3D human TS cultures was accompanied by spontaneous syncytialization, which appears to be a feature of growing primary CTBs and human TS cells as 3D cultures, regardless of the presence or absence of a matrix (Haider *et al.*, 2018; Turco *et al.*, 2018; Sheridan *et al.*, 2021; Yang *et al.*, 2022, 2024; Hori *et al.*, 2024). While CTB markers were detected throughout 3D human TS cultures, STB formation appeared to be primarily localized to the periphery. This distribution of trophoblast subpopulations is consistent with previous data from our lab (Jeyarajah *et al.*, 2022). Because these 3D cultures were maintained in stem media, we did not expect complete coverage along the exterior and were unable to confirm whether a continuous STB layer had formed. However, it was recently reported that reducing Wnt activation and increasing the percentage of FBS promotes formation of a more complete outer STB layer in 3D matrix-free human TS cultures (Hori *et al.*, 2024). A similar effect may be achieved in our 3D cultures by adopting these culture conditions.

The commonly used choriocarcinoma-derived trophoblast cell line BeWo exhibited a partial STB-like response when cultured in 3D, whereas this response was not detected in HTR cells. This difference is perhaps not unexpected, as treatment with cAMP does not induce STB differentiation in 3D clonogenic HTR cultures (Nandi *et al.*, 2018), suggesting that STB-like changes may be difficult to detect in this cell line. In contrast, 3D BeWo cultures displayed increased expression of STB-associated genes and increased hCG by immunofluorescence, consistent with partial acquisition of an STB-like phenotype. These findings align with a recent report showing that 3D BeWo cultures formed in agarose moulds secrete increased levels of CGB, although substantial cell fusion required treatment with a cAMP agonist (Apodaca *et al.*, 2024). Thus, while 3D culture appears to promote some STB-associated features in BeWo cells, additional validation is needed to determine whether this reflects bona fide STB differentiation or a more limited endocrine-like response.

Our findings also suggest that STB in the 3D human TS matrix-free cultures retains characteristic functions. For instance, the increase in secreted hCG demonstrates that the STB formed in 3D cultures possesses endocrine functions, similar to other reported 3D matrix-free cultures (Yang *et al.*, 2022, 2024). Phase-contrast microscopy reveals bubble-like structures indicative of cell fusion and reminiscent of previously reported 3D STB human TS cultures (Okae *et al.*, 2018). We detected multinucleated CGB-positive aggregates along the periphery, but due to challenges associated with clearly resolving individual cell boundaries around the periphery, we were not able to quantify the extent of fusion. In future studies, this could be resolved by using a more direct evaluation of fusion through membrane capacitance (Yang *et al.*, 2024), or pooling cells that express different fluorescent proteins (Hori *et al.*, 2024).

Surprisingly, expression of some EVT markers was increased within 3D matrix-free human TS cultures, albeit with less concrete evidence than for the STB lineage. HLA-G-positive staining was detectable in 3D cultures, although these positive cells were relatively sparse and did not exhibit the same consistent distribution or staining intensity observed for STB markers. Most reports, including our previous work, indicate that EVT differentiation conditions are required to induce robust EVT differentiation within 3D trophoblast cultures (Haider *et al.*, 2018; Turco *et al.*, 2018; Jeyarajah *et al.*, 2022; Arutyunyan *et al.*, 2023). Trophoblast organoids derived from primary CTBs and human TS cells can form both lineages under stem conditions, but the proportion of STB precursors heavily outweighs that of EVTs (Shannon *et al.*, 2024). Interestingly, the proportion of HLA-G-positive cells is diminished in trophoblast organoids cultured in suspension compared to those embedded in Matrigel (Yang *et al.*, 2024). Sporadic cells positive for STB and EVT markers have also been detected on the surface of trophoblast organoids cultured on collagen beads (Zhou *et al.*, 2025). While 3D matrix-free trophoblast cultures may display enhanced potential to form EVTs, this could reflect cells capable of following STB and EVT pathways.

The Hippo pathway was identified as an enriched term among genes downregulated in 3D cultures. Several of these genes are associated with YAP1/TAZ-TEAD transcriptional activity, prompting us to examine Hippo pathway-associated gene expression in 2D and 3D human TS cultures. *TEAD2* and *TEAD4* expression was reduced in 3D cultures, consistent with their decline during STB differentiation (Mizutani *et al.*, 2022). Notably, approximately half of the Hippo pathway genes differentially expressed between 2D and 3D cultures overlapped with genes differentially expressed following *TEAD4* knockdown in human TS cells. Of this subset, the directionality is mostly consistent between 3D matrix-free and *TEAD4* knockdown human TS cultures (Saha *et al.*, 2020). Additionally, many of the Hippo-related genes differentially expressed in 3D cultures are also TEAD4 targets in 2D human TS cells (Saha *et al.*, 2020). As such, reduced *TEAD4* expression in 3D cultures is consistent with canonical Hippo pathway activation and consequent suppression of YAP1/TAZ-TEAD signaling. Overall, our transcriptome data suggest that the Hippo pathway is activated in 3D matrix-free human TS cultures.

Inhibition of LATS1/2 with LATS-IN-1 led to decreased expression of STB markers in 3D human TS cultures, accompanied by morphological changes indicative of reduced fusion. These findings are consistent with previous reports showing that constitutive YAP1 activation reduces STB marker expression in 2D primary CTBs, human TS cells, and BeWo choriocarcinoma cells (Meinhardt *et al.*, 2020; Mizutani *et al.*, 2022). However, the relationship between LATS-IN-1 treatment, *TEAD4* expression, and STB differentiation was not straightforward. Although LATS-IN-1 reduced CGB transcript and protein levels, *TEAD4* transcript levels were not increased, despite evidence that LATS-IN-1 enhanced TEAD transcriptional activity. This suggests that LATS-IN-1 may suppress spontaneous STB differentiation through increased YAP1/TAZ-TEAD activity rather than through changes in *TEAD4* transcript abundance. Indeed, inhibition of LATS1/2 would be expected to reduce YAP1 phosphorylation, promote YAP1 nuclear localization, and enhance TEAD-dependent transcriptional output. Thus, 3D culture may reduce *TEAD4* expression as part of an early shift away from the CTB progenitor state, whereas LATS-IN-1 maintains YAP1/TAZ-TEAD activity and cell proliferation, thereby limiting the spontaneous syncytialization normally observed in 3D cultures. While further validation of Hippo pathway components is required in LATS-IN-1-treated 3D cultures, these findings suggest that reduced YAP1/TAZ-TEAD activity may be an important step for spontaneous STB formation in 3D matrix-free human TS cultures.

One fundamental limitation of using human TS cells is their alignment with proximal cell column progenitors in the EVT route instead of CTBs (Sheridan *et al.*, 2021; Shannon *et al.*, 2024). Accordingly, future studies should compare 3D matrix-free cultures derived from human TS cells with those made from primary CTBs. Nevertheless, our findings provide a foundation for understanding how 3D matrix-free trophoblast cultures differ from traditional 2D cultures. Moreover, the anatomically relevant organization of CTBs and STBs in the 3D matrix-free cultures may help overcome limitations associated with matrix-embedded trophoblast organoids for studies of placental barrier function (Karvas *et al.*, 2022; Yang *et al.*, 2022; Wu *et al.*, 2023). With further refinement, 3D matrix-free human TS cultures may provide a tractable platform for investigating trophoblast responses to established and emerging pathogens, as well as for assessing the transfer of drugs and metabolites across trophoblast barriers.

The present study provides a detailed investigation of trophoblast subpopulation identity and differentiation pathways within 3D matrix-free cultures. In this system, human TS cells preferentially differentiated toward STB along the periphery. Our findings also suggest that reduced YAP1/TAZ-TEAD signaling may contribute to spontaneous STB formation in 3D TS cultures. Overall, this study supports 3D matrix-free trophoblast cultures as feasible models for investigating placental biology and advancing fetal-maternal health research.

## Supplementary Material

gaag054_Supplementary_Data

## Data Availability

The RNA-sequencing datasets generated and analyzed in this study have been deposited in the Gene Expression Omnibus (GEO) database using accession number GSE294076. The data underlying this article will be shared on reasonable request to the corresponding author.
