# Supplementary material for: Hippo signaling regulates spontaneous human trophoblast syncytialization in three-dimensional cultures
Source: Mol Hum Reprod. 2026 Sep 8;32(4):gaag054. doi: 10.1093/molehr/gaag054 (PMC13641821; doi:10.1093/molehr/gaag054)
Supplement: Supplementary file 1 [file gaag054_supplementary_data.zip › Supplementary Information-post adjudication clean.pdf]

Supplementary information (SI)

Hippo signaling regulates spontaneous human trophoblast syncytialization in three-dimensional cultures

Michael Zheng, Lin Zhao, Richard Xi, Jahdiel DeNobrega, Mary Tammo, Stephen J. Renaud, Peeyush K. Lala

Table of contents:

Supplementary Figure S1: Differentiation of human trophoblast stem cells into syncytiotrophoblast and extravillous trophoblast lineages. 2

Supplementary Figure S2: Three-dimensional culture alters trophoblast subpopulation marker expression in human trophoblast stem cells. 3

Supplementary Figure S3: Unprocessed Western blots corresponding to images presented in Figures 1 and 5. 5

Supplementary Figure S4: Three-dimensional culture does not induce syncytiotrophoblast-like features in HTR-8/SVneo cells. 7

Supplementary Figure S5: LATS1/2 inhibition diminishes syncytiotrophoblast formation in three-dimensional matrix-free trophoblast stem cultures. 8

Supplementary Table S1: Forward and reverse primers for quantitative RT-PCR. 10

Other supplementary material not included in this PDF file:

Supplementary Table S2: RNA-sequencing dataset (separate Excel file)

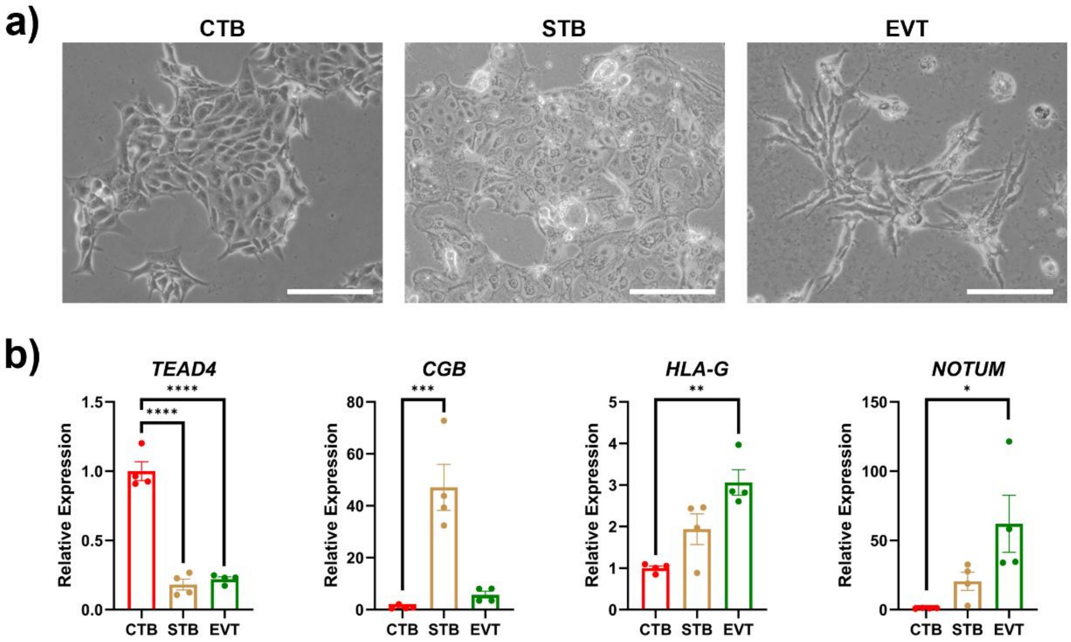

Supplementary Figure S1: Differentiation of human trophoblast stem cells into syncytiotrophoblast and extravillous trophoblast lineages.

Human trophoblast stem (TS) cells were maintained in complete TS media (cytotrophoblast; CTB) or placed in media promoting syncytiotrophoblast (STB) or extravillous trophoblast (EVT) differentiation. **a.** Phase-contrast images. The scale bars equal 200 μm. **b.** Quantitative RT-PCR analysis for CTB (*TEAD4*), STB (*CGB*), and EVT (*HLA-G*, *NOTUM*) markers. Graphs show mean ± SEM. One-way ANOVA with Tukey's post-hoc test for multiple comparisons. **P* < 0.05, ***P* < 0.01, ****P* < 0.001, *****P* < 0.0001; n=4 independent experiments.

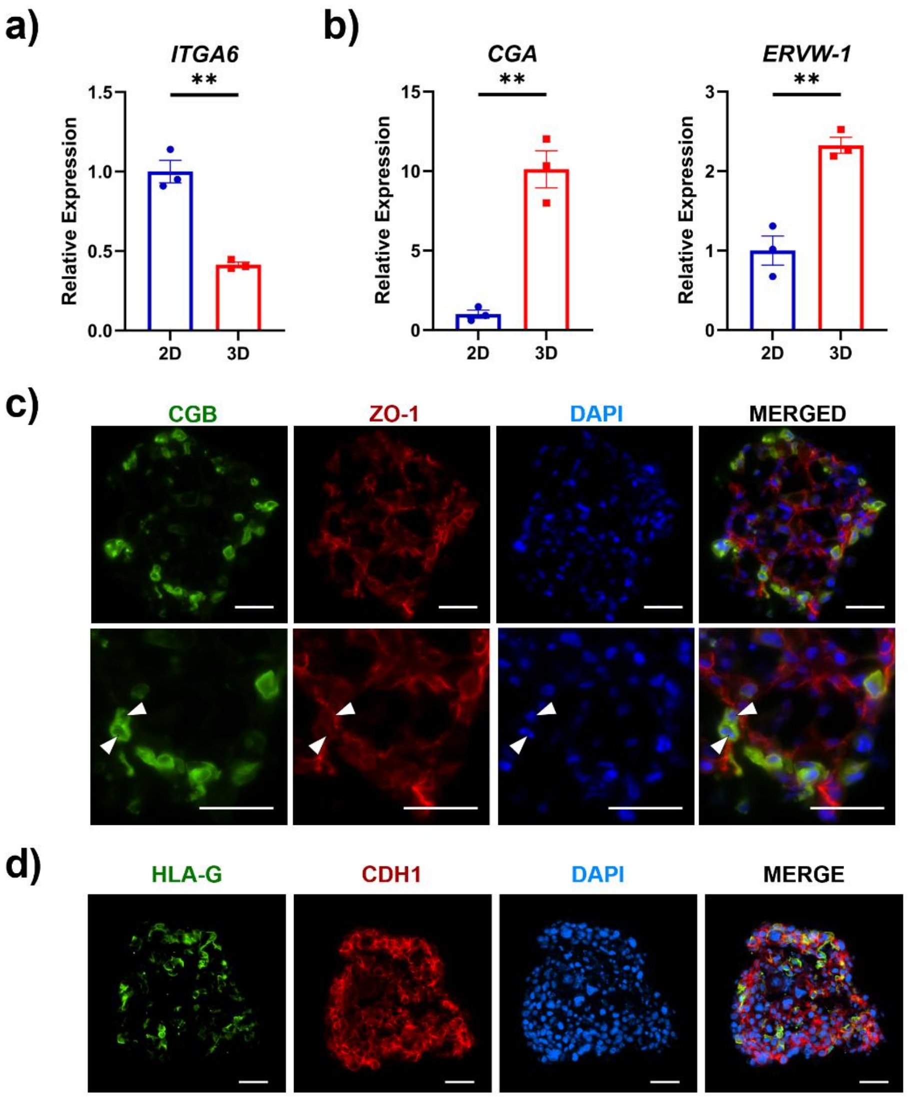

Supplementary Figure S2: Three-dimensional culture alters trophoblast subpopulation marker expression in human trophoblast stem cells.

Two-dimensional (2D) and three-dimensional (3D) human trophoblast stem (TS) cultures were grown in complete TS media. **a and b.** Quantitative RT-PCR analysis for the (a) cytotrophoblast (CTB) marker *ITGA6*, and the (b) syncytiotrophoblast (STB) markers *CGA* and *ERVW-1*. Graphs show mean ± SEM. Student's t-test. $**P < 0.01$; n=3 independent experiments. **c.** Immunofluorescence for CGB (green) and ZO-1 (demarcates cell junctions; red). The nuclei were stained with DAPI (blue). Higher magnification images are shown in the bottom panels. The arrowheads point to CGB-positive regions containing multiple nuclei with reduced ZO-1 staining,

indicative of STB formation. The scale bars equal 50 μm. **d.** Immunofluorescence for HLA-G (EVT marker) and CDH1. The nuclei were stained with DAPI (blue). The scale bars equal 50 μm.

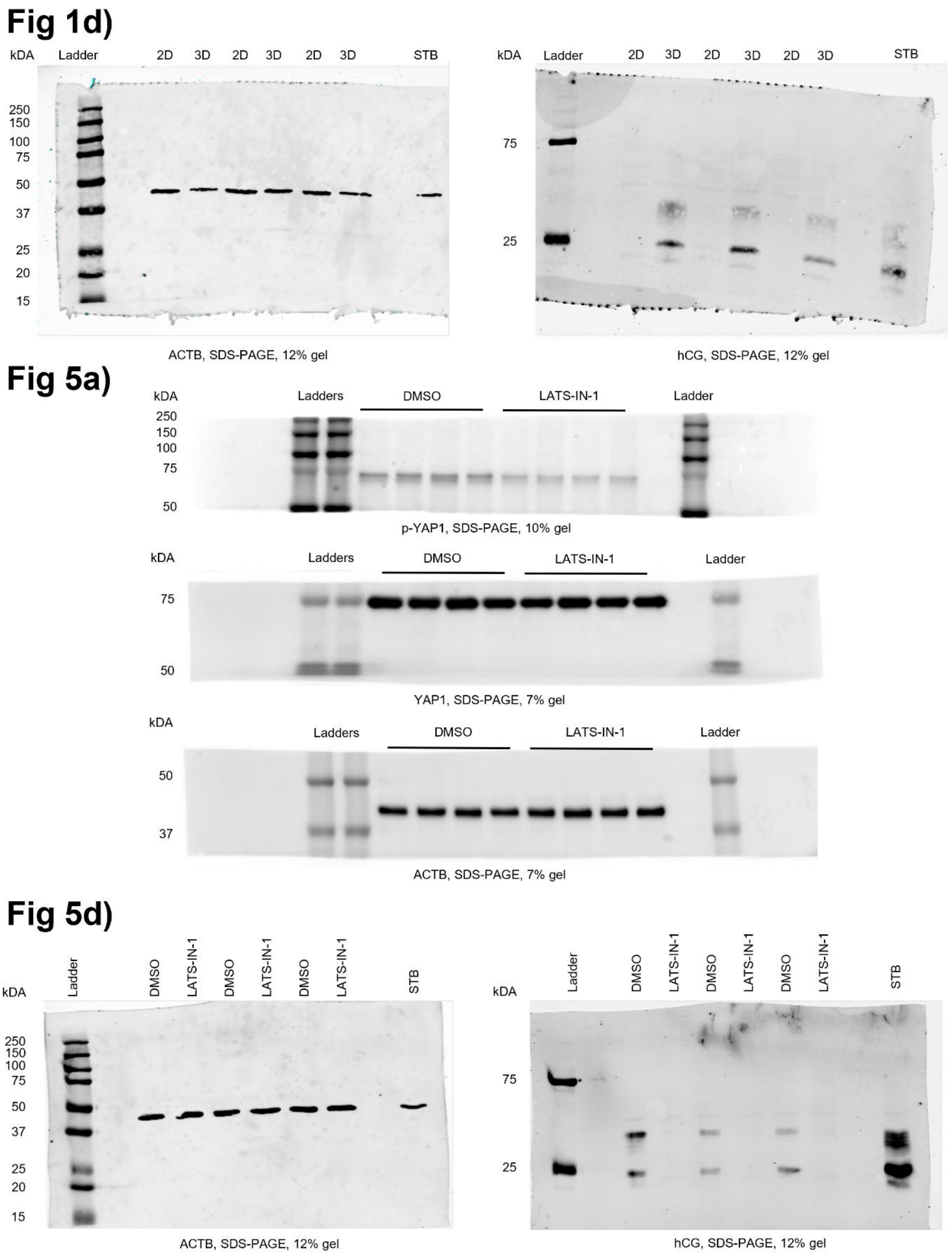

Supplementary Figure S3: Unprocessed Western blots corresponding to images presented in Figures 1 and 5.

Figure 1 samples include two-dimensional (2D) and three-dimensional (3D) human trophoblast stem (TS) cultures. Figure 5 samples include 3D human TS cultures treated with DMSO and LATS-IN-1. 2D syncytiotrophoblast (STB) cultures were used as controls.

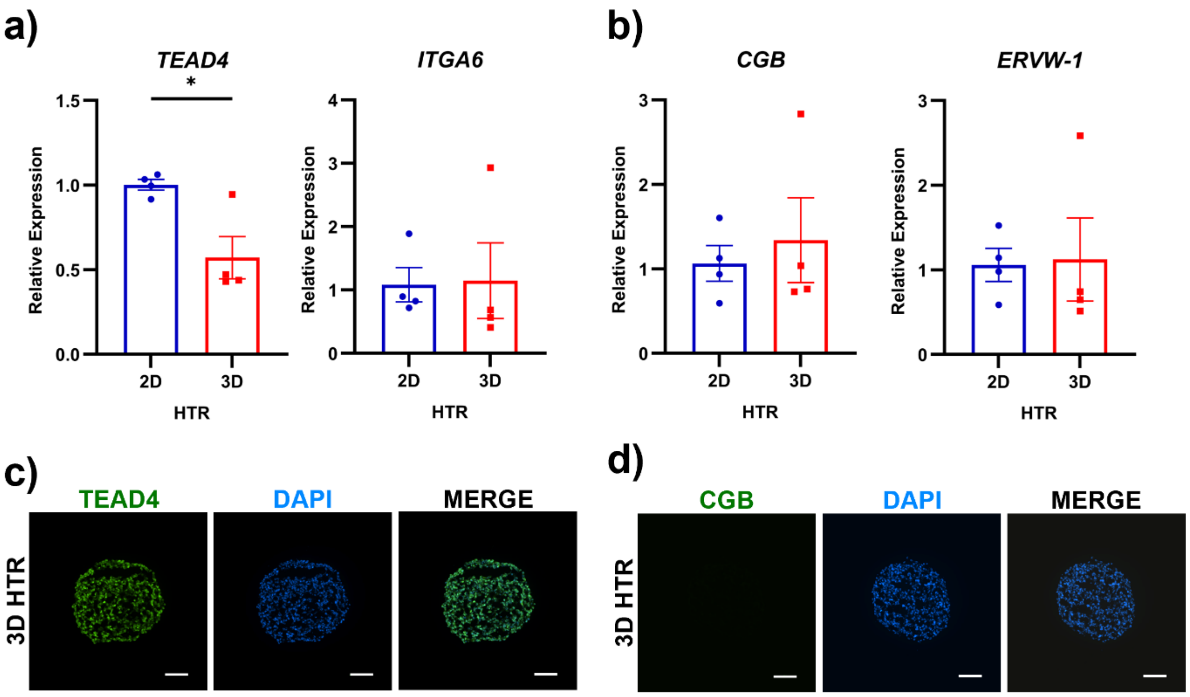

Supplementary Figure S4: Three-dimensional culture does not induce syncytiotrophoblast-like features in HTR-8/SVneo cells.

a and b. Quantitative RT-PCR analysis for (a) the cytotrophoblast (CTB) markers *TEAD4* and *ITGA6*, and (b) the syncytiotrophoblast (STB) markers *CGB* and *ERVW-1* between two-dimensional (2D) and three-dimensional (3D) HTR-8/SVneo (HTR) cultures. Graphs show mean ± SEM. Student's t-test. $*P < 0.05$; n=4 independent experiments. **c and d.** Immunofluorescence for (c) TEAD4 and (d) CGB in 3D HTR cultures. Nuclei were stained with DAPI. Please note that CGB was not detectable in HTR cultures. The scale bars equal 50 µm.

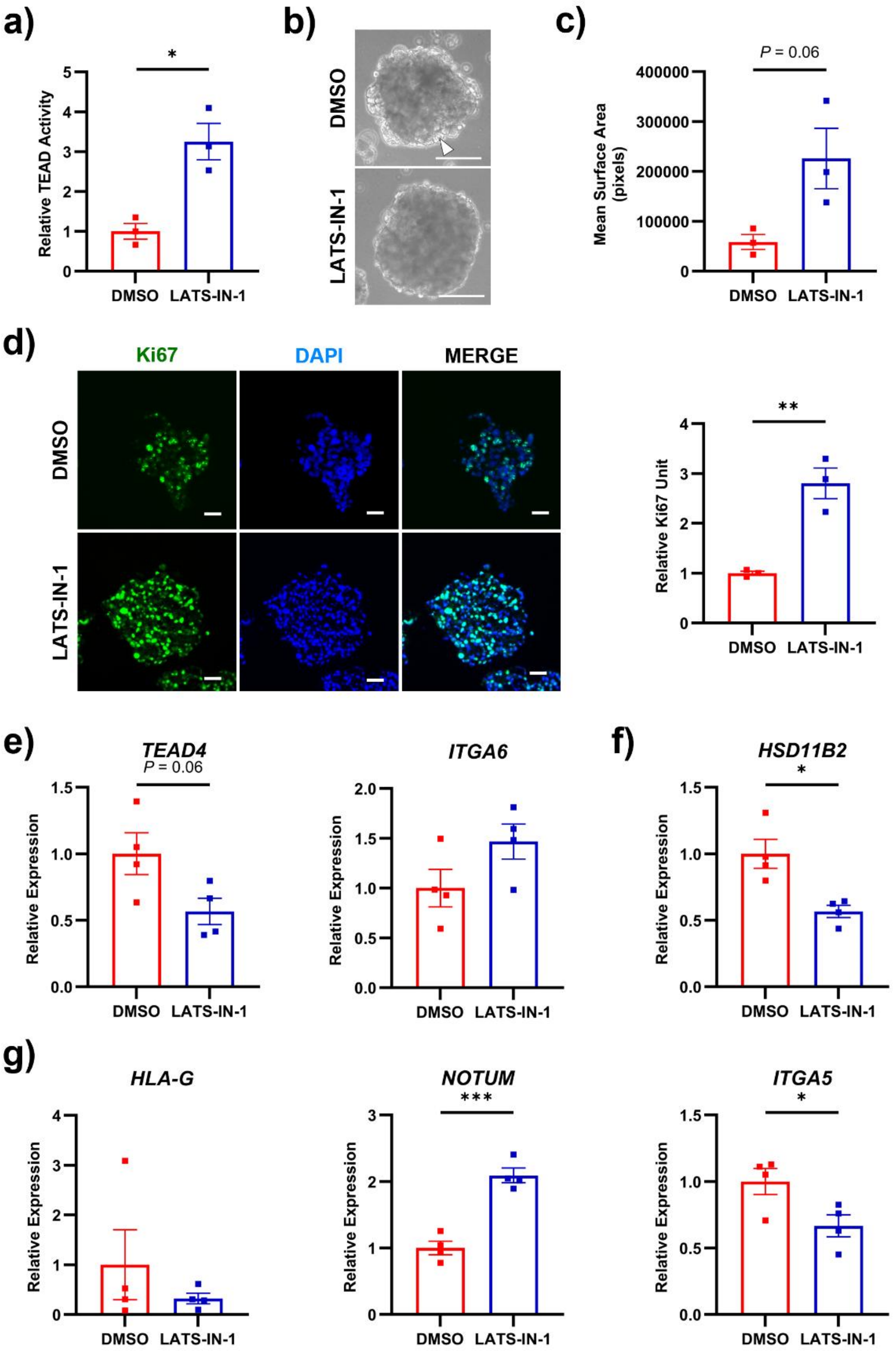

Supplementary Figure S5: LATS1/2 inhibition diminishes syncytiotrophoblast formation in three-dimensional matrix-free trophoblast stem cultures.

a. Two-dimensional (2D) human trophoblast stem (TS) cultures were grown in complete TS media containing DMSO or LATS-IN-1 (10 μM) and transfected with a plasmid encoding firefly luciferase under a YAP1/TAZ-responsive promoter; n=3 independent experiments. **b.** Phase-contrast images of three-dimensional (3D) human TS cultures grown in complete TS media treated with DMSO or LATS-IN-1, with white arrows for bubble-like morphology, indicative of syncytiotrophoblast (STB) formation. The scale bars equal 200 µm **c.** Quantification of mean surface area; n=3 independent experiments. **d.** Immunofluorescence for Ki67 in 3D human TS cultures treated with DMSO or LATS-IN-1. Nuclei were stained with DAPI. The scale bars equal 50 μm. The relative number of Ki67-positive cells is shown to the right; n=3 independent experiments. **e-g.** Quantitative RT-PCR analysis of (e) cytotrophoblast (CTB)-associated markers (*TEAD4* and *ITGA6*); (f) an STB-associated marker (*HSD11B2*); and (g) extravillous trophoblast (EVT)-associated markers (*HLA-G, NOTUM*, and *ITGA5*); n=4 independent experiments. Graphs show mean ± SEM. Student's t-test. $^*P < 0.05$, $^{**}P < 0.01$, $^{***}P < 0.001$.

Supplementary Table S1: Forward and reverse primers for quantitative RT-PCR.

**Gene**	**Accession No.**	**Forward Primer (5'–3')**	**Reverse Primer (5'–3')**
CGA	NM_001252383.2	GATCCACAGTCAACCGCCCT	TCTGGGCAATCCTGCACATC
CGB	NM_000737.3	CCTGGCCTTGTCTACCTCTT	GGCTTTATACCTCGGGGTTG
EEF2	NM_001961.4	GGCGTAGAACCGACCTTTG	GACAGCGAGGACAAGGACAA
ERVFRD-1	NM_207582.3	CCAAATTCCCTCCTCTCCTC	CGGGTGTTAGTTTGCTTGGT
ERVW-1	NM_001130925.2	CTACCCCAACTGCGGTTAAA	GGTTCCTTTGGCAGTATCCA
GAPDH	NM_001256799.3	AATGGGCAGCCGTTAGGAA	GCGCCCAATACGACCAAATC
HLA-G	NM_001363567.1	GCTACGGAATGAAGACGCCA	TACCTCATGGAGTGGGAGCC
HSD11B2	NM_000196.4	CAGATGGACCTGACCAAACC	AGCTCCGCATCAGCAACTAC
ITGA5	NM_002205.5	GCTTCAACTTAGACGCGGAG	CCCCAAGGACAGAGGTAGAC
ITGA6	NM_000210.4	GCAGCCTTCAACTTGGACAC	TCCCGTTCTGTTGGCTCTCT
MKI67	NM_002417.5	GAAGAGCTCCTAGCAGTCG	GGCCACTTCTTCATTCCAG
NOTUM	NM_178493.6	GCGACTCCAGATACGACACC	GACCATGTTTGCGTTCCACC
TEAD4	NM_003213.4	CAGTATGAGAGCCCCGAGAA	TGCTTGAGCTTGTGGATGAA
YWHAZ	NM_003406.3	ATGCAACCAACACATCCTATC	GCATTATTAGCGTGCTGTCTT
